# Characterisation of a divergent progenitor cell sub-populations in human osteoarthritic cartilage: the role of telomere erosion and replicative senescence

**DOI:** 10.1038/srep41421

**Published:** 2017-02-02

**Authors:** Christopher R. Fellows, Rebecca Williams, Iwan R. Davies, Kajal Gohil, Duncan M. Baird, John Fairclough, Paul Rooney, Charles W. Archer, Ilyas M. Khan

**Affiliations:** 1 Cardiff University, School of Biosciences, Cardiff, UK; 2 Centre for NanoHealth, Swansea University Medical School, Swansea, UK; 3 University of Surrey, Department of Veterinary Preclinincal Sciences, School of Veterinary Medicine, Faculty of Health and Medical Sciences, Guildford, UK; 4 Cardiff University, Institute of Cancer and Genetics, Cardiff, UK; 5 University Hospital of Wales, Cardiff, UK; 6 NHS Blood and Transplant Tissue Services, Liverpool, UK

## Abstract

In recent years it has become increasingly clear that articular cartilage harbours a viable pool of progenitor cells and interest has focussed on their role during development and disease. Analysis of progenitor numbers using fluorescence-activated sorting techniques has resulted in wide-ranging estimates, which may be the result of context-dependent expression of cell surface markers. We have used a colony-forming assay to reliably determine chondroprogenitor numbers in normal and osteoarthritic cartilage where we observed a 2-fold increase in diseased tissue (*P * < *0.0001*). Intriguingly, cell kinetic analysis of clonal isolates derived from single and multiple donors of osteoarthritic cartilage revealed the presence of a divergent progenitor subpopulation characterised by an early senescent phenotype. Divergent sub-populations displayed increased senescence-associated β–galactosidase activity, lower average telomere lengths but retained the capacity to undergo multi-lineage differentiation. Osteoarthritis is an age-related disease and cellular senescence is predicted to be a significant component of the pathological process. This study shows that although early senescence is an inherent property of a subset of activated progenitors, there is also a pool of progenitors with extended viability and regenerative potential residing within osteoarthritic cartilage.

Osteoarthritis (OA) is the progressive loss of normal joint function and is the world’s leading cause of physical disability in adults. In the UK 20% of adults aged between 50–59 have symptomatic disease in one or both knee joints, and this rises to 50% for people aged between 80–89^1^. The disease is mainly characterised by the degeneration and loss of articular cartilage leading to pain, joint stiffness and immobility. Treatments for osteoarthritis generally involve managing pain, inflammatory episodes, physiotherapy and lifestyle modifications in order to slow the progression of disease. When conservative forms of therapy are exhausted, patients can elect to have artificial joint replacement surgery to relieve pain and regain some mobility. The question of whether, in the earliest stages of disease, it is possible to stimulate repair in order to spare the joint rests on the presence of responsive cellular components. In general, tissue repair and regeneration requires a viable pool of stem or progenitor cells, but, in the case of articular cartilage the relative quiescence of chondrocytes originally led biologists to assume that all the cells in this tissue were terminally differentiated and post-mitotic^2^. The latter notion fitted the observations that once diagnosed joint lesions invariably led to osteoarthritis of the whole joint. The discovery of progenitor cells in articular cartilage has led to a re-evaluation of possibilities for stimulating endogenous repair and for their use for tissue engineering applications, and not least, in studying disease mechanisms^3^,^4^,^5^. In the latter case, understanding the fundamental properties of chondroprogenitor cells such as the number of resident progenitors and whether they constitute a reservoir of cells in normal or diseased cartilage able to respond to reparative stimuli, may provide the basis of developing new therapeutic interventions.

Colony forming ability is a well-recognised trait of stem/progenitor cells and has been used extensively to perform quantitative and functional analysis of clonal populations of progenitors. In the absence of cell-specific biomarkers, differential adhesion of chondrocytes to fibronectin has proved to be an extremely useful method to enrich and isolate clonogenic progenitor populations from bovine, equine and human articular cartilage^3^,^4^,^6^,^7^. The assay is based on two observations; the presence of bromodeoxyuridine label retaining cells in the surface of articular cartilage^5^,^8^, and, work showing the surface of fetal knee cartilage is rich in fibronectin, correlating with the presence of alphaV integrins in superficial zone chondrocytes^9^. In addition to their colony forming ability fibronectin-enriched cells are positive for CD90, CD105, CD166, STRO-1, Notch-1, negative for CD45 and CD34 cell surface markers and are able to undergo multi-lineage differentiation, and thus fulfill the minimal criteria to be classified as multipotent mesenchymal stromal cells (MSC)^4^. Clonal isolates of bovine and human articular chondroprogenitors have been shown to continually express SOX9 and exhibit telomere maintenance after extensive culture expansion, and also retain a capacity for re-differentiation^7^,^10^. Moreover, articular chondroprogenitors unlike marrow stromal mesenchymal cells do not generate a collagen type X-rich matrix or express RUNX2 transcription factor protein upon chondrogenic differentiation, but produce an extracellular matrix that is predominantly hyaline in nature^6^,^7^. Other methods to isolate chondroprogenitors with MSC attributes primarily using fluorescence-activated cell sorting techniques, have been described^3^,^11^,^12^, but, the estimates of progenitors frequency exhibiting antibody reactivity to MSC cell surface markers and displaying multipotential plasticity fall within a wide range of between 3–17%, making reliable estimation of progenitors in cartilage tissues a challenging prospect. Therefore, using a differential adhesion colony-forming assay for the measurement of progenitor numbers and method to isolate clonal populations for downstream analyses, should provide a more accurate and coherent picture of their status in normal and diseased cartilage. The importance of understanding progenitor cell characteristics is critical if strategies such as autologous chondrocyte implantation and stimulation of intrinsic repair are to be effectively applied. In this study, we demonstrate that colony-forming chondroprogenitors from osteoarthritic cartilage are composed of two distinct sub-populations based on their proliferative capacity, telomere length analysis and senescence profiles when compared to cells from normal tissue. We discuss the possible origins of the divergent progenitor sub-population, and, how our findings impact on the development of intrinsic and extrinsic cartilage repair mechanisms.

## Results

**Figure 1:**
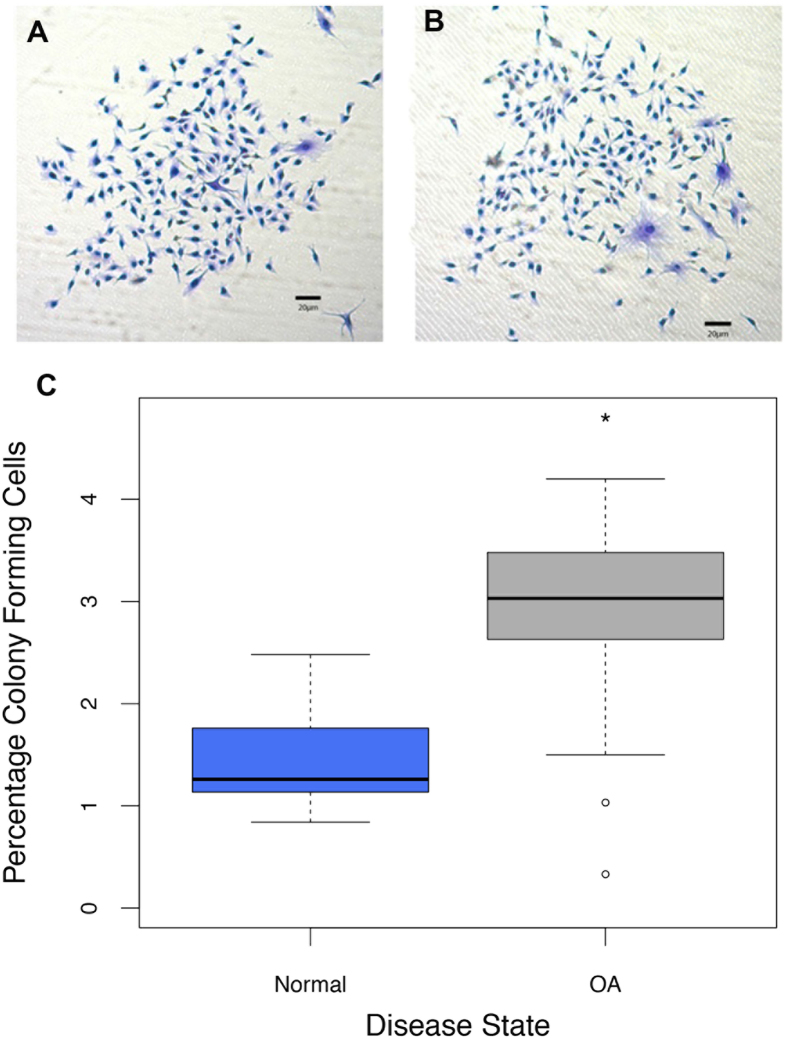
Clonogenic adult progenitor cells were isolated from normal and osteoarthritic cartilage. (**A**,**B**) Fibronectin-adhered colonies from normal (N-CPC) and osteoarthritic (OA-CPC) cartilage were stained with crystal violet dye. (**C**) Number of colonies were quantified based on initial seeding density, box plots demonstrate the percentage mean of clonogenic cells from OA cartilage digests (n = 11) was 2.80% ( ±0.29%) compared to 1.47% ( ±0.16%) for normal cartilage digests (n = 11). The approximately 2-fold increase was statistically significant (*) using T-test (P = 0.0001).

The ability of adult stem cells to undergo self-renewal allows for their identification by a standard colony-forming assay. Articular cartilage-derived chondroprogenitor (CPC) cells from human normal or osteoarthritic cartilage were enriched from their differentiated progeny by differential adhesion to fibronectin. Resulting colonies were stained by crystal violet and quantified as a percentage of the initial seeding density for each tissue type, Fig. 1A–C. There was approximately a 2-fold increase (T_22_ = −4.58; P = 0.0001) in the mean percentage of fibronectin-adhered cell colonies derived from osteoarthritic cartilage (2.80 ± 0.29%; n = 11) when compared to normal cartilage (1.47 ± 0.16%; n = 11), (Fig. 1C).

**Figure 2:**
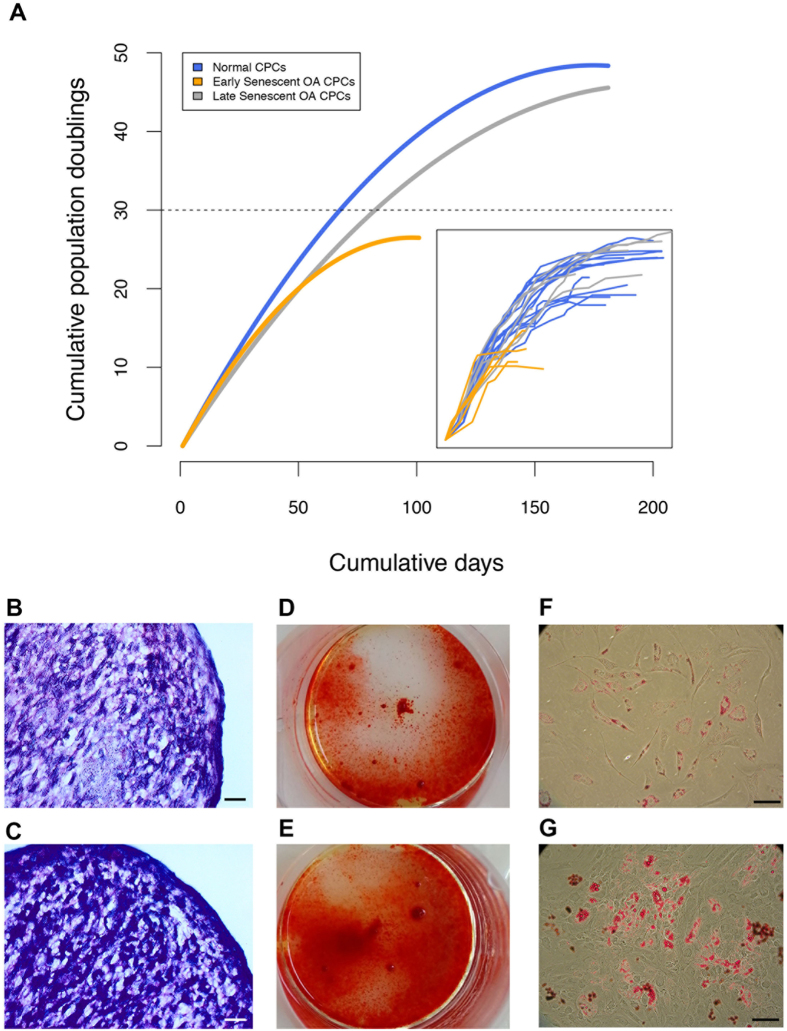
OA cartilage consists of divergent populations of adult stem cells. (**A**) Non-linear regression curves of the average growth kinetics (population doublings) of Normal (n = 17) and OA-derived (n = 21) clonogenic cell lines. OA-CPC cell lines diverged into two groups, early senescent (grey line) and late senescent (orange line). N-CPCs consisted of a single cohort that was capable of prolonged proliferation (blue line). *Inset*; Individual clonal cell line growth kinetics. (**B**,**C**) Representative images for chondrogenic differentiation of LS-OA-CPCs (**B**) and ES-CPCs. (**C**). Chondrogenic 3D pellets were stained with toluidine blue. (**D**,**E**) Representative images for osteogenic monolayer differentiation of LS-OA-CPCs (**C**) and ES-CPCs. (**D**). Mineralisation was stained using alizarin red. (**F**,**G**) Representative images for adipogenic monolayer differentiation of LS-OA-CPCs (**F**) and ES-CPCs. (**G**). Lipid accumulation was stained using oil red O. Scale bars 100 μm.

Growth kinetics (population doublings) of the clonally-derived chondroprogenitor cell lines from a subset of 6 normal and 7 OA donors were investigated (Fig. 2A). A total of 17 monoclonal cell lines were cultured from normal cartilage (N-CPCs) and 21 monoclonal cell lines from OA cartilage (OA-CPCs) (Fig. 2A-Inset). The initial growth rates of CPCs were found to be linear until approximately 30 PD after which the rate of proliferation started to slow. The most distinguishing feature is that OA-derived cell lines can be sub-divided into two populations, an early senescent population (ES-OA-CPCs) and a late-senescent population (LS-OA-CPCs). Of the 21 OA-CPC lines, 11 (52.3%) senesced before reaching 30 PD, the remainder displayed growth rates comparable to stem cells from normal cartilage. The ratio of ES-CPCs to LS-CPCs is shown for each patient in Supplementary Table 1.

CPCs from both normal and OA cartilage have previously been shown to have tri-lineage potential^7^,^13^. To confirm both the ES-OA-CPCs and LS-OA-CPCs were capable of phenotypic plasticity, chondrogenic, osteogenic and adipogenic differentiation was induced in both types of CPC (Fig. 2B–G). Both early and late senescent OA subpopulations were capable of tri-lineage differentiation.

**Figure 3:**
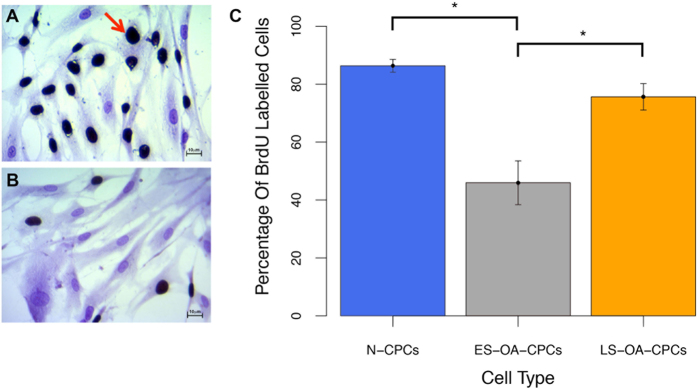
BrdU labelling with positive cells shown with dark nuclei, the red arrow indicates a characteristic positive cell. Cells were counterstained with crystal violet. (**A**,**B**) Representative images of BrdU labelling in a late senescent cell line (**A**) and early senescent cell line (**B**). (**C**) Percentage of BrdU positive cells. ES-OA-SC lines analysed demonstrated a significantly reduced proliferative index (*) compared to N-CPCs (p = 3.1 × 10^−6^) and LS-OA-CPCs (p = 0.0005) (Anova with Tukey’s HSD pair-wise comparisons). Error bars represent the standard error of the mean. Scale bars 10 μm.

**Figure 4:**
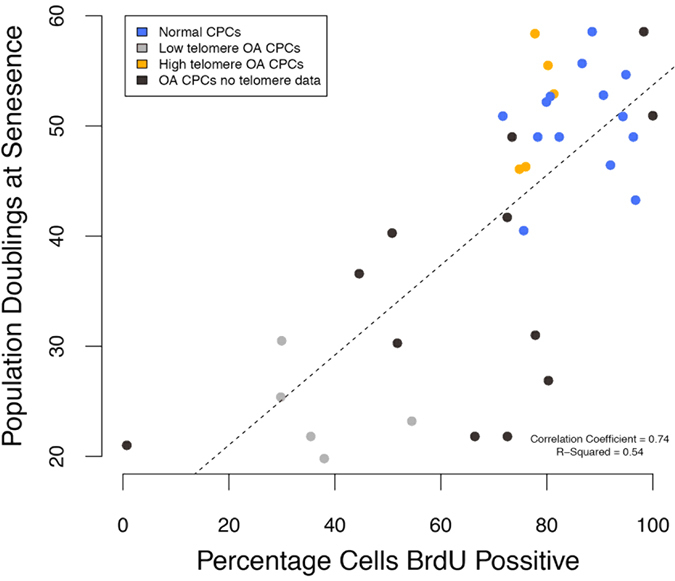
Scatter plot and linear regression showing a statistically significant correlation between population doublings at time of senescence and the percentage BrdU positive cells at 22–25 population doublings (p = 3.07 × 10^−4^). Pearson’s product-moment correlation = 0.74. Blue points show N-CPC samples (long telomeres), orange points show OA-CPCs that had long telomeres, grey points show OA-CPCs with short telomeres and black points show OA-CPCs for which no telomere data was available. Long telomere samples have prolonged replicative and proliferative capacity as indicated by population doublings and BrdU.

**Figure 5:**
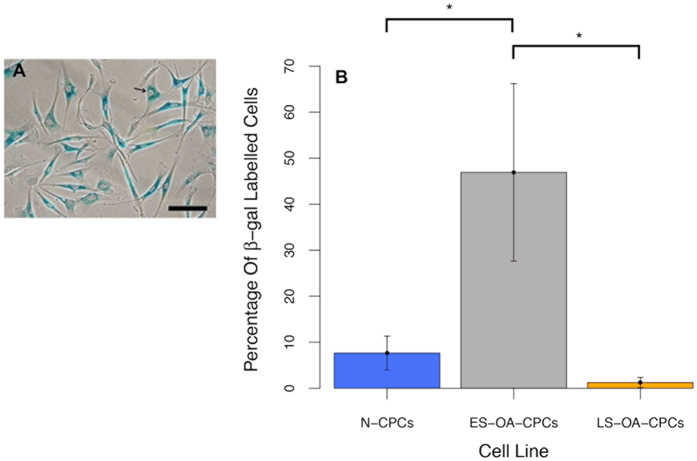
(**A**) Representative image for positive SA-βgal labelling. (**B**) CPCs lines were analysed for senescence indices. A 38.3-fold increase in SA-βgal positive cells was observed in early senescent OA CPCs compared to late senescent OA CPCs (p = 0.029) and a 6.1-fold increase compared with normal CPCs (p = 0.032). Statistical significance (*) was shown using Anova with Tukey’s HSD pair-wise comparisons. Error bars represent the standard error of the mean.

To confirm the variation observed in the growth kinetics of OA-CPCs the proliferation and senescence indices of clonogenic isolates were investigated. Early senescing OA-derived CPC isolates display a lower proliferative index, measured through bromodeoxyuridine (BrdU) labelling index, than either normal (F_1,33_ = 18.41; p = 3.1 × 10^−6^) or late-senescent OA CPC isolates (F_1,33_ = 18.41; p = 0.0005) (Fig. 3A–C). At 22–25 PD early senescent OA-CPCs displayed an average of 46.0% proliferating cells whereas normal and late-senescent cell lines had an average of 86.4% and 75.6% proliferative cells respectively. BrdU labelling was found to significantly correlate with the number of population doublings at senescence (correlation coefficient = 0.74; F_1,34_ = 40.27; p = 3.07 × 10^−4^), (Fig. 4). Early senescing clonal isolates also exhibited 38.3-fold greater labelling (46.9 ± 19.3%; n = 6) for SA-βgal activity compared to late senescent OA isolates (1.2 ± 1.1%; n = 5) (F_2,17_ = 5.169; p = 0.029), and 6.1-fold more labelling than clonogenic isolates from normal cartilage (7.6 ± 3.7% n = 9), (F_2,17_ = 5.169; p = 0.032), (Fig. 5A,B).

**Figure 6:**
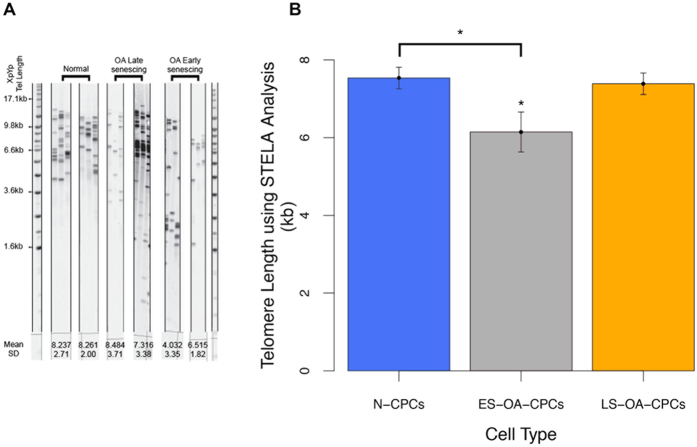
Single Telomere Length Analysis (STELA) of the 17p and XpYp telomeres. (**A**) Representative gels for 2 samples from each sample group. Normal CPCs (n = 6) uniformly display high telomere length. In contrast, OA-derived CPCs (n = 10) show either low (OA early senescing) or high (OA late senescing) telomere lengths. (**B**) Average telomere length (kb) of N-CPCs, ES-OA-CPCs and LS-OA-CPCs. ES-OA-CPCs have significantly shorter telomeres that N-CPCs (p = 0.047). ES-OA-CPCs also have a non-significant decrease in telomere length compared to LS-OA-CPCs (p = 0.093). Statistical significance (*) was shown using Anova with Tukey’s HSD pair-wise comparisons. Error bars represent the standard error of the mean. Individual sample lanes are cropped to provide representative samples, full gel image is provided in the Supplementary Information.

**Figure 7:**
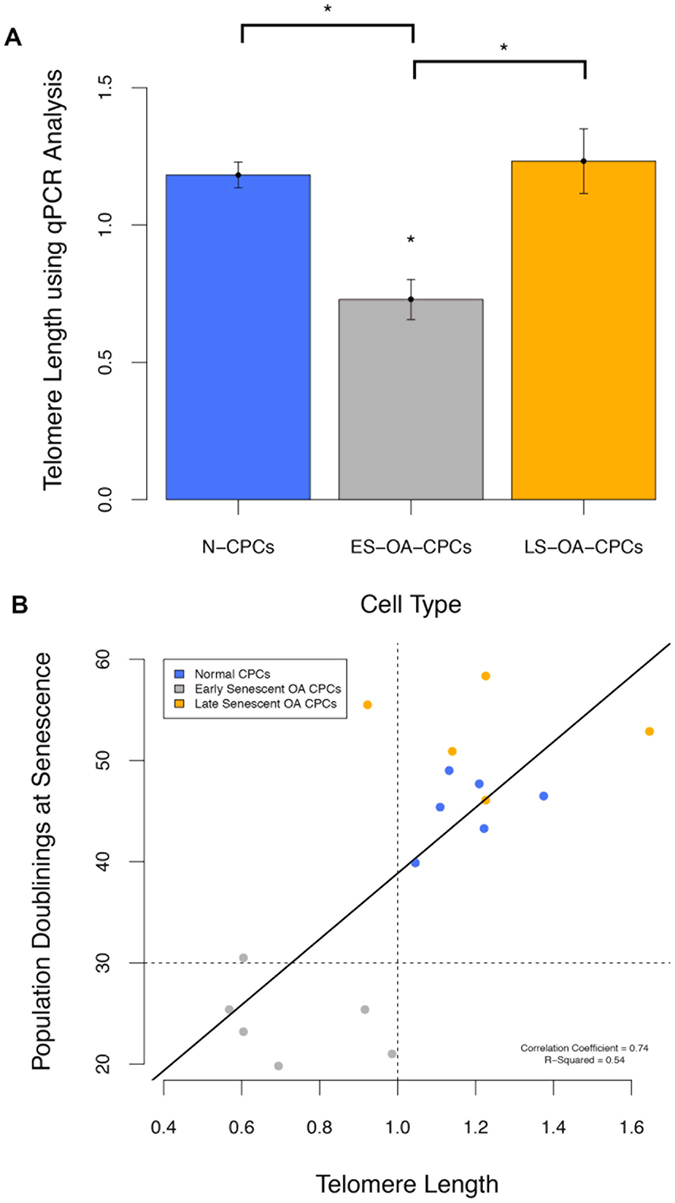
The average telomere length of all chromosomes was investigated using qPCR. (**A**) Telomere length was 38% lower in ES-OA-CPCs compared to N-CPCs (p = 0.0026) and 41% shorter compared with LS-OACPCs (p = 0.0017). Statistical significance (*) was shown using Anova with Tukey’s HSD pair-wise comparisons. (**B**) Average telomere length at 22–25 population doublings correlates with the number population doubling at senescence. Pearson’s product-moment correlation coefficient = 0.74, (p = 7.2 × 10^−4^). Shorter telomeres are predictive of ES-OA-CPCs and longer telomeres predictive of LS-OA-CPCs.

The replicative potential of OA-derived CPCs correlates with their chromosomal telomere length. High-resolution analysis of telomeres using STELA of representative clonal isolates from the three distinct colony-derived cell populations clearly show that early senescent OA-isolates have lower average single telomere ends (6.15 ± 0.51) than either N-CPCs (7.54 ± 0.67) (F_2,13_ = 4.121; p = 0.047) or LS-OA-CPCs (7.38 ± 0.62) (F_2,13_ = 4.121; p = 0.093) (Fig. 6A,B). Using a rapid qPCR based assay to detect the average telomere length of all chromosomes, the relative telomere lengths of ES-OA-CPCs (0.73 ± 0.07) were 38% shorter compared to normal clonal isolates (1.18 ± 0.04) (F_1,14_ = 12.39; p = 0.0026) and 41% shorter than LS-OA-CPCs (1.23 ± 0.12) (F_1,14_ = 12.39; p = 0.0017) (Fig. 7A). Pre-senescence telomere length (PD22-25) was found to correlate with the number of population doubling at the time of senescence (Correlation coefficient = 0.74; F_1,15_ = 17.96; p = 7.2 × 10^−4^), (Fig. 7B). Shorter telomeres were predictive of ES-OA-CPCs and longer telomeres predictive of LS-OA-CPCs. Cell lines that were used for both BrdU analysis and telomere length analysis showed a direct correlation between short telomeres and lowered BrdU labelling index (Fig. 4).

## Discussion

In this study, we focused on the isolation and quantification of articular cartilage-derived progenitor cells from normal and OA human knee joints and compared their characteristics with particular focus on proliferative potential and telomere dynamics. Our data shows that in contrast to progenitors isolated from normal human cartilage, OA-derived progenitors are present at an increased frequency and consist of two divergent subpopulations that can be separated on the basis of proliferative potential and capacity for telomere maintenance. We discuss the possible origin of divergent progenitor populations in diseased cartilage and the implications of their presence in terms of pathological development of disease.

Using differential adhesion to fibronectin to enrich CPCs from their differentiated progeny, we found that OA cartilage has on average 2-fold more clonogenic progenitor cells compared to normal cartilage. Two studies that used fluorescence-activated cell sorting for cell surface markers CD105 and CD166 have also shown progenitors are present in normal and diseased human cartilage with different frequencies (3.5% normal and 8% OA^3^; 15% normal and 17% OA^12^). However, the latter studies used cultured chondrocytes (passages 0–1) and there is strong evidence showing levels of cell surface markers change significantly upon adherence to plastic, thus complicating analysis^11^,^14^. Diaz-Romero and colleagues (2008) have analysed the phenotypic plasticity of cells on plastic in terms of changes in the levels of MSC-related cell surface proteins in human articular chondrocytes (HAC) compared to bone marrow-derived MSCs^15^. They found that although both cell types exhibit a similar surface antigen profile, levels of CD166 (P0-P1, 24-fold), CD90 (P0-P1, 390-fold), CD49c (P0-P1, 19-fold) and CD44 (P0-P1, 4-fold) increased in HACs following culture but remained relatively unchanged in marrow-derived MSCs, indicating rapid adoption of a more naïve, dedifferentiated phenotype by HACs following culture expansion^15^. The implication is that dedifferentiated chondrocytes either mask the presence of true progenitors or that dedifferentiated chondrocytes are de facto progenitors. Richardson (2011) showed that freshly isolated bovine chondrocytes are positive for CD105 labelling but, it is only after 14 days in culture that a discrete brighter sub-population (1.37%) emerges^16^. That study also showed evidence of chondrocyte dedifferentiation through unimodal distributions of CD166, CD29 and CD49e. The results of cell sorting using surface antigens are therefore dependent on many context dependent variables that can lead to multiple inferences. In contrast, colony forming assays do not suffer from the same problem relying instead on progenitors’ ability to bind to fibronectin and their capacity to replicate beyond 5 PD (>32 cells), in order to distinguish them from transit amplifying cells. Colony forming assays are therefore a more representative and reproducible method of quantifying progenitor cell number^4^.

The reasons for increases in progenitor frequency in OA cartilage found in this study are probably the result of either enforced self-renewal or cellular recruitment from the immediate environment. Mankin showed that cellular proliferation measured by radiolabelled thymidine incorporation rose proportionally with histological-histochemical grading of osteoarthritic tissue until a crisis point is reached at Grade 10 (Mankin Grade scales from 0–15)^17^. Beyond Mankin Grade 10 labelling fell rapidly up to a point when measurements were not possible due to tissue degradation (Grade 13/15). Mankin and colleagues also showed that DNA levels remain essentially constant until overt tissue loss, suggesting the presence of homeostatic mechanisms that potentially regulate cell number through activation of quiescent progenitors in the tissue. Multiple studies have shown that biomarkers of foetal and immature chondrocytes are re-expressed in osteoarthritic tissue including procollagen type IIA^18^,^19^ and atypical chondroitin sulphate epitopes such as 3B3(−) and 7D4^20^,^21^, lending support to the notion, in the absence of more specific biomarkers, that chondroprogenitor cells are being amplified. On the other hand, recruitment of stem cells into cartilage to maintain homeostasis and replace lost cells during injury and disease is hypothesised to occur through migration of stem cells from the synovium^22^, node of Ranvier^23^, bone marrow^24^, or the synovial fluid^25^. The work of Jones *et al*. showed a 7-fold increase in synovial fluid MSCs during early OA and speculated on their origin as being synovium^25^. Koelling demonstrated that migratory MSCs could be isolated from late stage OA tissue with some evidence indicating these cells originate from the bone marrow and enter cartilage through breaks in the tidemark^24^. Whether migratory MSCs actually aid or impair cartilage repair is debateable because although these cells are capable of chondrogenic differentiation *in vitro* there is no evidence that the same process is active *in situ*. Similarly, there is no evidence for engraftment of stem cells into intact articular cartilage, so it is more likely, that at least during the earliest phases of injury and disease, chondroprogenitor proliferation is the primary pathway of repopulating the tissue^26^.

The increase in progenitor frequency is concurrent with the emergence of a divergent OA progenitor subpopulation. Upon culture expansion OA-CPCs could be separated into two groups; one that underwent replicative exhaustion by 30 PD and classified as early-senescent (ES-CPC), and another that was capable of prolonged expansion or late-senescent (LS-CPC). Chondroprogenitor cell lines from a healthy donor proliferated at similar rates with a small degree of variation between different donors, whereas OA-derived CPCs from single donors displayed a large degree of variation in proliferation kinetics. A study by Nelson and colleagues (2014) also identified that 50% of the progenitor cell lines established from OA cartilage failed to reach 30 PD, however it is unclear whether this variation was within cell lines from the same patient or between donors of a wide age range^13^. We found early and late senescent progenitors could be isolated from the same donor with no difference in the frequency of subpopulations as a function of donor age (p = 0.56). Other studies have also shown no correlation between the frequency of progenitors in cartilage and donor age, sex, or OA grade^3^. Therefore, we believe the divergent progenitor subpopulations are a facet of OA pathophysiology.

Why divergent populations arise is probably related to the fundamental properties of stem/progenitor cells. Stem cells balance self-renewal and differentiation through asymmetric or symmetric divisions where cell fates, stem or committed daughter, are regulated through feedback mechanisms directed by the cellular microenvironment to maintain homeostasis^27^. Disturbances in the microenvironment can adversely affect progenitor cell homeostasis, and in cartilage a conceptual framework for how this occurs through dissolution and loss of a putative stem cell niche has been proposed by Hayes *et al*.^28^. We hypothesise that cell-intrinsic factors reduce the replicative potential of daughter cells, which still display cell surface determinants of progenitors. Tissue-specific progenitors with different replicative potential have been identified in many tissues, such as normal epidermis, where three hierarchical clonal populations; holoclones, meroclones and paraclones, (in order of colony forming ability) can be isolated^29^. Experiments have shown the proportion of holoclones in skin declines with age whilst that of the paraclones rises. We did not see age-dependent divergence in behaviour in progenitors derived from cartilage isolated from donors who did not have clinical symptoms of OA (oldest donor 85 years of age) so we believe that the difference in replicative potential of OA-derived CPCs is a consequence of disease. In addition, whilst the clonogenic ability of cells declines in aging skin, this does not happen in either normal or OA cartilage, probably because progenitors in cartilage are relatively more quiescent that those in epidermis^7^,^13^.

The major difference between the OA-derived divergent species is their replicative potential, with early and late senescent clonal cell lines able to be isolated from the same donor. The identifying feature of the ES-CPCs is a reduced proliferation rate and increased telomere erosion compared to similarly passaged cell lines. The replicative potential of cells has been shown to have direct physiological consequences as shown by Hayflick, where the fibroblasts’ ability to undergo cellular division negatively correlates with the age of the donor^30^. The replicative potential of stem/progenitor cells is important for long-term maintenance of implanted tissue engineered cartilage and also to reduce the incidence of age-related disease^31^. Therefore, we predict the presence of progenitor sub-populations has two importance pathological consequences in diseased cartilage, first, as an indicator that differentiation is impeded, and secondly, that non-differentiated progenitor clones may increasingly populate the matrix, and exacerbate the lack of ECM maintenance by undergoing senescence following replicative senescence. Our previous work has shown that human chondroprogenitors undergo active telomere maintenance during culture expansion and display increasing telomerase activity in line with the appearance of a subset of cells displaying high average telomere length^7^. In this study we again employed a high resolution single chromosomal telomere analysis, STELA^32^ to show that ES-CPC can be retrospectively distinguished from LS-CPCs derived from normal and OA cartilage. ES-CPCs lost on average 1.2 kbp more telomeric repeats after 22 PD–25 PD compared to LS-CPCs. The *in vivo* telomeric erosion rate is estimated to be 30 bp/year for chondrocytes, therefore the difference between ES and LS clones equates to 40 years of aging^31^. Given the increased proliferation rate within diseased cartilage up to Mankin Grade 10, it is not unreasonable to propose that senescent cells would start to increasingly populate this cartilage. Senescent cells also have been identified in cartilage using SA-βgal activity mainly in the proximity of osteoarthritic lesions^33^. Studies have also shown that alterations in the secretome of chondrocytes occurs upon senescence with increased production of pro-inflammatory cytokines and matrix metalloproteinases^34^. Therefore, deranged progenitors that have adopted a senescence-associated secretory phenotype following replicative exhaustion may be a significant contributor to the progressive degradation of cartilage within the osteoarthritic knee. Telomere erosion through replication is not the only mechanism for the induction of senescence; oxidative damage, mitochondrial dysfunction and stress-induced senescence will also contribute to this phenotype^35^,^36^.

In conclusion, our work has shown that progenitors are not only present within osteoarthritic cartilage but their frequency is increased. Our data also shows that a divergent sub-population of the OA-derived progenitors have reduced proliferative potential and undergo early senescence *in vitro*. The presence of deranged progenitors in cartilage will eventually result in increased numbers of senescent cells causing long-term deleterious effects. Determining if divergence of progenitor characteristics occurs either as a consequence of cell-intrinsic or extrinsic changes will require experimental verification. Experiments will also have to test the possibility that divergent sub-populations may be derived from migrating MSCs from either subchondral bone^24^ or synovium^37^. Crucially, the remaining population of late-senescing progenitors are capable of chondrogenic differentiation and may be a viable pool of cells to activate regeneration and repair of the remaining cartilage. To mobilise these cells and initiate productive repair of osteoarthritic lesions, a combination of cellular reprogramming and recapitulation of the normal stem cell niche may be necessary. If we can resolve these issues in future research, the potential for endogenous repair of osteoarthritic lesions will be a realistic goal.

## Methods

### Tissue and cell isolation

Full-depth normal human articular cartilage samples (n = 11; mean age 55.6 yrs, range 25–85, IQR 36.5–72.5) from deceased donors and cartilage from patients undergoing total knee replacements for osteoarthritis (n = 11; mean age 66.8 yrs, range 54–85, IQR 59–72) were obtained with fully informed patient consent and in accordance with local NHS Research Ethics Committee guidelines. South East Wales NHS Research Ethics Committee specifically approved this study and all experimental protocols were performed in accordance with the relevant guidelines and regulations (09-WSE04/35). Cartilage biopsies (6 mm^2^) from both normal and diseased tissue were excised from the tibial plateau, diced and chondrocytes isolated by a sequential pronase (70 U ml^−1^, 1 hour at 37 °C; 11459643001, Roche) and collagenase (300 U ml^−1^, 3 hours at 37 °C; C0130, Sigma) digest. Biopsies from OA donors were taken from the lateral aspect of the tibial plateau from a region adjacent to cartilage lesions. This area showed macroscopic roughening but was not fully degraded. Seven of the OA cartilage biopsies were histologically graded using the modified Histological-Histochemical Grading System (mean score 3.25, range 1–6, IQR 2.5–4). Normal cartilage biopsies were taken from the corresponding region of macroscopically undamaged cartilage from non-symptomatic donors.

### Fibronectin adhesion assay and chondroprogenitor cell isolation

Isolated cells were subjected to a fibronectin adhesion assay to identify colony forming chondroprogenitor cells^7^. Six well plates were coated with 10 μg.ml^−1^ fibronectin (Sigma, UK, F1141) in 0.1 M phosphate buffered saline (PBS, pH7.4) containing 1 mM MgCl_2_ and 1 mM CaCl_2_ overnight at 4 °C. Isolated full-depth chondrocytes (500 cells in 1 ml) were seeded onto the fibronectin coated plates for 20 mins at 37 °C in Dulbecco’s modified Eagle’s medium (DMEM) (Gibco, UK, 41965-062), after which media and non-adherent cells were removed and replaced with fresh DMEM containing penicillin 100 μg.ml^−1^/streptomycin 100 U.ml^−1^ (Gibco, UK, 15140-122), 0.1 mM ascorbic acid (Sigma, UK, A8960), 0.5 mg.ml^−1^ L-glucose (Sigma, UK, G6152), 10 mM HEPES pH7.4 (Gibco, UK, 15630-056), 1 mM sodium pyruvate (Gibco, UK, 11360-070), 2 mM L-glutamine (Gibco, UK, 25030-081) and 10% foetal bovine serum (FBS) (Gibco, UK, 10106-169 1). This media was termed DMEM+. The remaining adherent cells which were maintained in culture for up to 12 days. The seeding density (500 cells/well) and the numbers of colonies (>32 cells) formed by day 12 were recorded to calculate percentage of progenitor cells in each tissue sample. Differential adhesion assays were preformed in triplicate for each sample. Data were analysed using a Student’s T-test.

### Progenitor colony expansion

At day 12, colonies were stochastically chosen, selected using cloning rings, and expanded in DMEM+ containing 1 ng.ml^−1^ transforming growth factor-β2 (PeproTech, UK, 100-35B) and 5 ng.ml^−1^ fibroblast growth factor-2 (PeproTech, UK, 00-18B). At each passage, the number of cells harvested and re-plated were recorded to calculate population doublings (PDs) over time^38^. Growth kinetics (population doublings) of the clonally-derived chondroprogenitor cell lines from a subset of 6 normal and 7 OA donors were investigated for long term culture. Cell lines were cultured until senescence; senescence was defined as no cell divisions over a 2-week period.

### Proliferative and senescence Index

At 22–25 PDs, (passage 8–9) culture-expanded CPC cell lines were plated at 3000 cells/cm^2^ and cultured for 72 hrs, the final 24 hrs in the presence of 10μM BrdU. Cultures were fixed in 95% ethanol (EtOH) for 10 mins, washed in PBS-Tween (0.1%) and then labelled with anti-BrdU antibodies at 1:20 dilution (G3G4, DHSB, University of Ohio) using the Vectastain Universal Quick kit (Vectorlabs, UK). DAB peroxidase substrate kit (Vectorlabs, UK) was used to visualise positively staining nuclei. Cells were then counter stained with 0.05% crystal violet for 30 minutes. A subset of 9 normal (3 donors) and 11 OA CPCs (4 donors) were analysed for senescence quantified through analysis of the labelling index with senescence-associated β-galactosidase (SA-βgal) activity as previously described^39^. In brief, cells were washed twice in PBS, fixed with 3% formaldehyde in PBS for 3 min, washed twice in PBS, and then stained for 18 h at 37 °C with fresh galactosidase staining solution. The solution contained 1 mg. ml^−1^ 5-bromo-4-chloro-3-indolyl-D-galactoside (X-Gal) in dimethylforamide, 40 mM citric acid/sodium phosphate, 5 mM potassium ferrocyanide, 5 mM potassium ferricyanide, 150 mM NaCl, 2 mM MgCl_2_, pH 6.0. After staining, cells were washed twice with PBS. BrdU and SA-βgal positive cells were counted and calculated as a percentage of the total number of cells on the plate – a minimum of 400 cells were counted per sample, and the data analysed using ANOVA, with post hoc pair-wise analyses performed using Tukey’s HSD.

### Differentiation

Differentiation studies were undertaken to determine phenotypic plasticity. Chondrogenic differentiation was performed in pellet culture, half a million cells were placed in a 1.5 ml Eppendorf tube containing 1 ml of chondrogenic media containing DMEM/F12 plus Glutamax with 10% heat-inactivated FBS, penicillin 100 μg.ml^−1^/streptomycin 100 U. ml^−1^, 100 μg. ml^−1^ ascorbic acid, 1 mg. ml^−1^ L-glucose, 2 mM L-glutamine, 1% HEPES, and supplemented with 1% insulin-transferring-selenium (ITS), 0.1 μM dexamethasone and 10 ng.ml^−1^ TGF-β1. Cells was pelleted at 2000 rpm for 10 minutes and incubated at 37 °C and fed every second day for 3 weeks. Osteogenic and adipogenic differentiation was undertaken in monolayer. Six-well plates were seeded at 5 × 10^4^ cells per well and cultured in standard culture media until 70–80% confluent. Media was changed to the appropriate differentiation media for 10 days with the media changed every 2 days. Osteogenic differentiation medium contained DMEM plus 10% FCS, 10 mM β-glycerophosphate, 10 nM dexamethasone and 0.1 mM L-ascorbic-acid-2-phosphate, penicillin 100 μg.ml-1/streptomycin 100 U.ml^−1^ and 1% HEPES. Adipogenic differentiation media contained DMEM plus 10% FCS containing 10 μg.ml^−1^ insulin, 1 μM dexamethasone, 100 μM indomethacin, 500 μM 3- isobutyl-1-methylxanthine (IBMX) and 15% normal rabbit serum.

### Telomere length analysis

Six Normal and 10 OA-derived CPC cell lines were subjected to single length telomere analysis (STELA) at the 17p and XpYp telomeres as described previously^40^. Average telomere length analysis was performed using an additional 6 normal and 11 OA-derived CPC cell lines using the qPCR-based method as described by Epel and colleagues^41^. The cell lines used for STELA and qPCR analyses were distinct. Telomere length analyses were performed on cell lines at 22–30 PD (passage 8–10).

### Statistics

All statistical tests were performed using the ‘R’ statistical package. Data sets were checked for normal distribution using the Shapiro-Wilks test. Tests for statistical significance were preformed using T-test and ANOVA with Tukey’s HSD pair-wise comparisons. Mean values are reported as ± standard error. Correlation coefficients were calculated using Pearson’s product-moment correlation analysis and linear regression. The average growth kinetic for each group was calculated as a best-fit curve from all samples within the group.

## Additional Information

**How to cite this article**: Fellows, C. R. *et al*. Characterisation of a divergent progenitor cell sub-populations in human osteoarthritic cartilage: the role of telomere erosion and replicative senescence. *Sci. Rep.* **7**, 41421; doi: 10.1038/srep41421 (2017).

**Publisher's note:** Springer Nature remains neutral with regard to jurisdictional claims in published maps and institutional affiliations.

## Supplementary Material

Supplementary Table 1 and Figure 1
