# Supplementary material for: Characterisation of a divergent progenitor cell sub-populations in human osteoarthritic cartilage: the role of telomere erosion and replicative senescence
Source: Sci Rep. 2017 Feb 2;7:41421. doi: 10.1038/srep41421 (PMC5288717; doi:10.1038/srep41421)
Supplement: Supplementary Table 1 and Figure 1 [file srep41421-s1.pdf]

Characterisation of a divergent progenitor cell sub-populations in human osteoarthritic cartilage: the role of telomere erosion and replicative senescence

*Dr Christopher R Fellows[1,2,3], *Dr Rebecca Williams[1], Dr Iwan R Davies[1], Dr Kajal Gohil[1], Prof Duncan M Baird[4], Prof John Fairclough[5], Dr Paul Rooney[5], Prof Charles W Archer [2], Dr Ilyas M Khan[2]

[1]Cardiff University, School of Biosciences, Cardiff, UK

[2] Centre for NanoHealth, Swansea University Medical School, Swansea, UK

[3] University of Surrey, Department of Veterinary Preclinincal Sciences, School of Veterinary Medicine, Faculty of Health and Medical Sciences, Guildford, UK

[4] Cardiff University, Institute of Cancer and Genetics, Cardiff, UK

[5] University Hospital of Wales, Cardiff, UK

[6] NHS Blood and Transplant Tissue Services, Liverpool, UK

*Both authors contributed equally to this work

Corresponding author – Ilyas M. Khan, PhD, Centre for NanoHealth, Swansea University Medical School, Singleton Park, Swansea, UK, SA2 8PP, Tel; 01792 602588, E-mail: I.M.Khan@swansea.ac.uk

Acknowledgements – This works was supported by Arthritis Research UK (Grant 20069) and Medical Research Council stem cell capacity building scholarship. CF is a member of the D-BOARD Consortium funded by European Commission Framework 7 programme (EU FP7; HEALTH.2012.2.4.5-2, project number 305815, Novel Diagnostics and Biomarkers for Early Identification of Chronic Inflammatory Joint Diseases). IK is supported by the UK Regenerative Medicine Platform.

Sample	Modified HHGS Score	ES-CPC cell lines established	LS-CPC cell lines established
OA 1	Unknown	3	0
OA 2	1	1	2
OA 3	6	1	2
OA 4	1	2	1
OA 5	3	2	1
OA 6	4	1	2
OA 7	4	1	2

Supplementary table 1: Table to indicate the modified Histological-Histochemical Grading Score for the OA cartilage samples used to generate CPC cell lines. The table indicated the number of early senescent (ES-CPC) and late senescent (LS-CPC) cell lines established from each donor

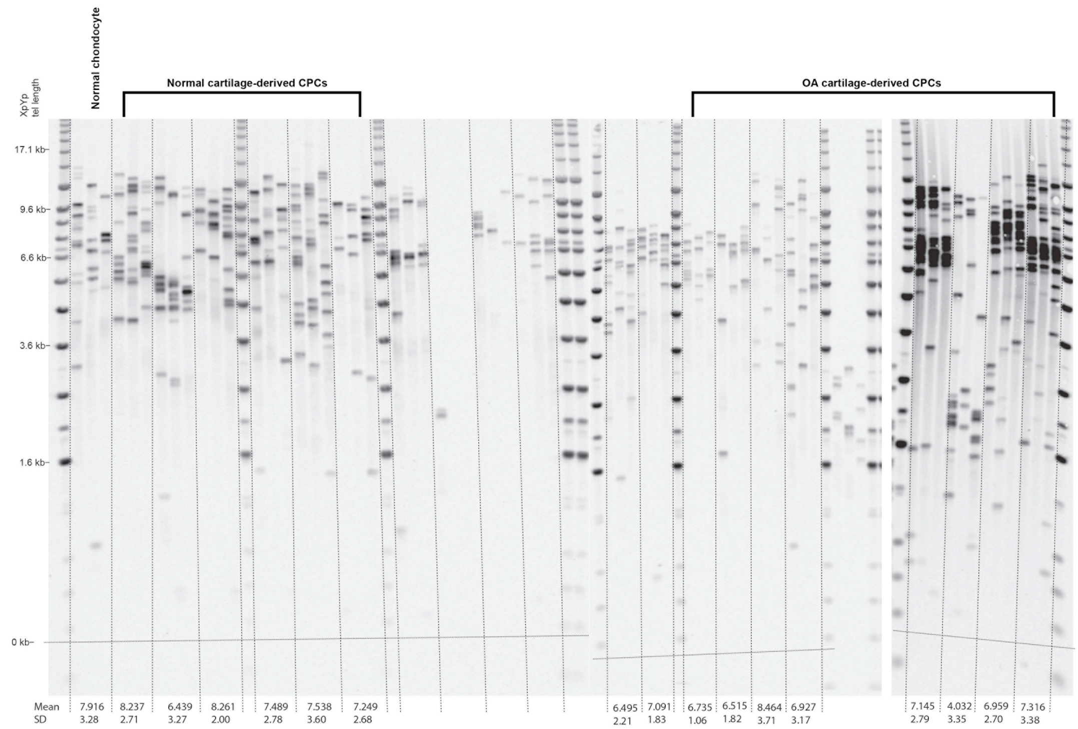

Supplementary figure 1: Single Telomere Length Analysis (STELA) of the 17p and XpYp telomeres. Normal CPCs (n=6) display high telomere length. In contrast, OA-derived CPCs (n=10) show either low (OA early senescing) or high (OA late senescing) telomere lengths.
